# Sugar and SPY(ce): Large-scale identification of SPINDLY-dependent *O*-fucosylation targets in Arabidopsis

**DOI:** 10.1093/plcell/koad024

**Published:** 2023-02-06

**Authors:** Sophie Hendrix

**Affiliations:** Assistant Features Editor, The Plant Cell, American Society of Plant Biologists, USA; Centre for Environmental Sciences, Hasselt University, Diepenbeek, Belgium

Besides their essential roles as substrates for carbon and energy metabolism, sugars also act in signal transduction, coordinating developmental and metabolic processes with nutrient availability. In animals, posttranslational modifications of nucleocytoplasmic proteins with β-*N*-acetylglucosamine (GlcNAc; an amide derivative of glucose) by *O*-GlcNac transferase (OGT) play a key role in nutrient sensing (Liu et al. 2015). The Arabidopsis (*Arabidopsis thaliana*) genome encodes two OGT homologs: SECRET AGENT (SEC) and SPINDLY (SPY) (Sun 2021). Whereas SEC is a canonical OGT, SPY catalyzes protein *O*-fucosylation using GDP-fucose (a nucleotide sugar derived from GDP-mannose) as a substrate.

SPY was initially identified as a negative regulator of gibberellin signaling and its importance for plant growth is underlined by the range of developmental defects observed in Arabidopsis *spy* mutants, which are generally more pronounced than those in *sec* mutants (Jacobsen and Olszewski 1993; Hartweck et al. 2002; Sun 2021). Recent studies identified a small number of SPY-interacting proteins, which were involved in hormone signaling, circadian clock function, as well as the regulation of transcription and RNA splicing. However, these only represented the tip of the iceberg among SPY targets, as has become apparent now.

In this issue of *The Plant Cell*, **Yang Bi, Ruben Shrestha and colleagues (Bi et al. 2023)** identified a large number of *O*-fucosylated proteins from Arabidopsis by affinity purification. The authors first showed that *spy* mutants grew poorly on sucrose-supplemented media in the dark (see Figure), indicating a crucial role for SPY in sugar-dependent growth. To identify targets of SPY-mediated *O*-fucosylation, they developed an affinity chromatography method utilizing *Aleuria aurantia* lectin (AAL), which specifically binds to *O*-fucose (Bandini et al. 2016). A proteome-wide screen in wild-type plants using AAL affinity chromatography followed by mass spectrometry identified 943 *O*-fucosylated peptides originating from 467 different proteins. *O*-fucosylated peptides were either undetectable or found in strongly reduced levels in *spy* mutants (see Figure), supporting the idea that SPY is the only protein *O*-fucosyltransferase catalyzing terminal *O*-fucosylation in Arabidopsis. According to the subcellular localization database SUBA4.0 (https://suba.live/), almost 67% of the identified proteins had a predicted nuclear localization, and only 12% and 9% were predicted to be localized in the cytosol and plastids, respectively. By contrast, *O*-fucosylation in metazoans is found only in secreted and cell surface proteins and is catalyzed by ER-localized *O*-fucosyltransferases (Sun 2021).

**Figure:**
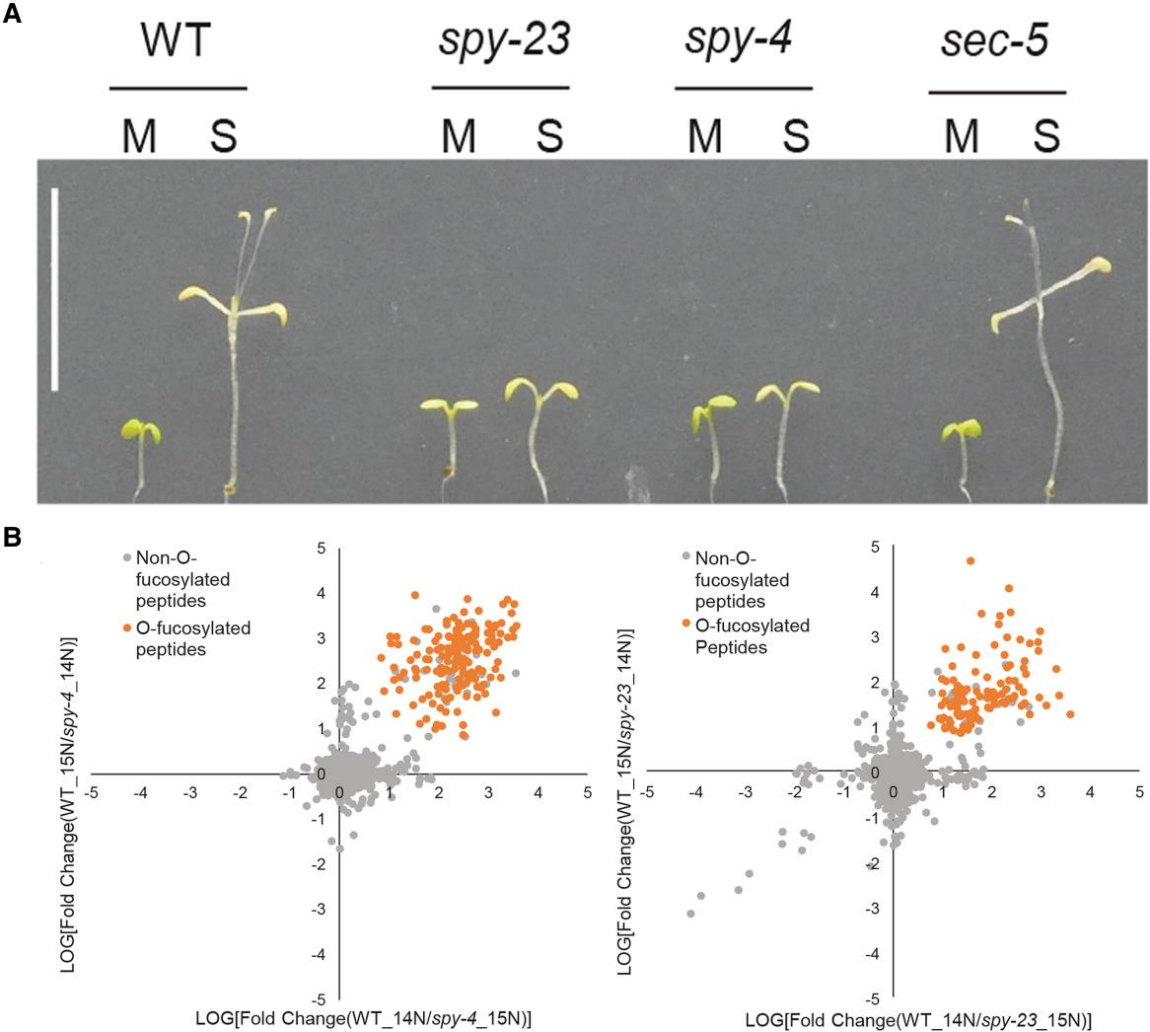
SPY mediates sugar-dependent growth and catalyzes protein *O*-fucosylation. A) Phenotype of WT, *spy* and *sec* mutant seedlings grown in the dark for eight days on media containing 1% mannitol (M) or sucrose (S). B) Scatter plots of log10 ratios of WT/*spy-4* (left) and WT/*spy-23* (right) for peptides detected and quantified in both isotope-switched replicate experiments. *O*-fucosylated peptides are shown in orange. Adapted from Bi et al. (2023, Figs. 1 and 2).

The results confirmed several previously reported SPY interactors including the DELLA protein RGA and the bHLH transcription factors TCP14 and TCP15, supporting the validity of the affinity chromatography approach. Gene ontology analysis showed significant enrichment of key molecular functions such as histone modification, transcription, RNA processing, translation, protein degradation and phosphorylation, the cytoskeleton, and vesicle trafficking among *O*-fucosylation targets. Interestingly, several of the identified proteins are also proposed targets of SEC and/or TARGET OF RAPAMYCIN (TOR), providing evidence for the integration of SPY signaling with other nutrient signaling pathways. The involvement of *O*-fucosylated proteins in key biological processes such as hormone signaling, light signaling, the circadian clock, root development, flowering, and immunity likely underlies the developmental and physiological defects seen in *spy* mutants, but whether and how protein *O*-fucosylation integrates these processes with the plant's energy status requires further investigation.

The large-scale identification of *O*-fucosylation targets in this work will undoubtedly pave the way for further studies on the physiological roles of the respective proteins, further improving our understanding of plant nutrient signaling. It will be interesting to determine how *O*-fucosylation influences the activity of the identified target proteins and whether *O*-fucosylation patterns change throughout plant development and in response to environmental stress. As previous work suggests that *O*-fucosylation and *O*-GlcNAcylation have antagonistic effects on some and additive effects on other target proteins, efforts should also be undertaken to further unravel the complex interactions between SEC and SPY signaling.
